# Cognitive styles and specific learning disorders in children and adolescents

**DOI:** 10.1192/j.eurpsy.2021.1031

**Published:** 2021-08-13

**Authors:** D. Galletta, M. D’Amaro, S. Celentano, C. Santoriello, R. Passerini

**Affiliations:** 1 Department Of Head-neck Care Unit Of Psychiatry And Psychology “federico Ii” University Hospital Naples, “Federico II” University Hospital Naples, Italy, Naples, Italy; 2 Sanitary Pole, LA FILANDA LARS, SARNO, Italy

**Keywords:** COGNITIVE STYLES, LEARNING DISORDERS, INTELLECTUAL FUNCTIONING, Working memory

## Abstract

**Introduction:**

Learning Difficulties relates to significant and unusual difficulties in the acquisition and use of one or more of the following areas: listening, speaking, reading, writing and mathematical skills. In the last twenty years, following the research conducted by cognitive psychology, from neuropsychology, from pedagogy and from the confrontation between educators and psychologists, the attention was focused on the cognitive modalities of the subjects engaged in learning tasks.

**Objectives:**

Thanks to the study of cognitive styles and Learning Styles thelearning subject was placed at the center of the educational project, stimulating from on the one hand there is also reflection on teaching styles and the most appropriate ones methodologies, teaching methods and methods of approaching the individual disciplines and, on the other hand, prompting clinicians to research around the intellectual peculiarities of each subject and a outline a descriptive criterion of his / her cognitive functioning profile.

**Methods:**

The intellectual scale (WISC IV) of 32 children (aged between 7 and 15 years) with specific learning disabilities was analyzed, in order to highlight the underlying intellectual functioning and any cognitive styles.

**Results:**

According to the international scientific literature, the results show a greater fall in the area of working memory, followed by the cognitive domain concerning processing speed.

**Conclusions:**

In detail, by analyzing the individual subtests, greater difficulties are noted, at all ages, in the processes of abstraction and conceptualization, in short-term auditory memory, in the speed of processing and visual-praxic motor coordination.

